# SNAP Work Requirements Reversal and Program Enrollment

**DOI:** 10.1001/jamahealthforum.2025.1587

**Published:** 2025-05-30

**Authors:** Hannah Factor, Jacob Wallace, Matthew Lavallee, Anthony Lollo, Chima D. Ndumele

**Affiliations:** 1 PhD Program in Health Policy, Harvard University, Cambridge, Massachusetts; 2 Yale School of Public Health, New Haven, Connecticut; 3 Department of Health Policy and Management, Johns Hopkins Bloomberg School of Public Health, Baltimore, Maryland

## Abstract

This cohort study assesses the association of implementation and reversal of Supplemental Nutritional Assistance Program (SNAP) work requirements with SNAP enrollment.

## Introduction

The US social safety net is an array of government programs designed to combat poverty by providing access to basic needs. In response to concerns that the availability of safety net benefits discourages people from working, the federal government and some states plan to expand work requirements for programs including the Supplemental Nutritional Assistance Program (SNAP) and Medicaid.^1^ These policies require able-bodied recipients to work or participate in a qualifying workforce program to maintain coverage. Prior research found that SNAP work requirements reduced short-term program participation and introduced additional administrative burdens, particularly for clinically and socially vulnerable households, but the lasting effects of this policy remain less understood.^2^

Given the potentially detrimental effects of work requirements, many policy observers have called for them to be repealed or narrowed.^3^ While such action may insulate against negative effects for new enrollees, it is unclear whether removing work requirements increases SNAP participation in the short term since households that lose coverage would need to be aware of the policy change and reapply.^4,5^ Understanding whether the effects of work requirements are reversible provides critical information to lawmakers weighing the long-term consequences of these policies. This cohort study examined a natural experiment wherein Connecticut imposed and then reversed SNAP work requirements for some residents but not others. We compared the enrollment gains from removing work requirements with the initial disenrollments when they were imposed.

## Methods

The primary data source was linked administrative data on Medicaid or SNAP enrollees in Connecticut between 2015 and 2018. Using US Department of Agriculture waiver data, we identified 87 towns with work requirements starting in January 2016; 41 eliminated work requirements in January 2017 (eFigure in Supplement 1). The full experiment is described elsewhere.^2^ This study was approved by the Yale University institutional review board and followed the STROBE reporting guideline. Informed consent was waived because the study used administrative data with no direct participant contact.

We estimated a triple-difference model that compared changes over time in SNAP enrollment for able-bodied adults without dependents in work requirement towns compared with exempt towns and with unaffected parents and caretakers. We estimated our triple-difference model separately in towns where work requirements were eliminated in January 2017 vs those where they were retained using an event study at the person-month level. To improve the comparability of groups, we matched individuals on characteristics in the Table.^6^ SEs were clustered at the town level, and statistical significance was assessed at *P < *.05. Analyses were performed from September 2023 to January 2025 using R, version 4.3.3.

**Table.** Sample Population of Adults in Connecticut as of March 2016

Characteristic	Targeted able-bodied adults without dependent, No. (%)^a^			Untargeted parents and caregivers, No. (%)^a^		
	Work requirements eliminated (n = 8490)	Work requirements remained (n = 10 682)	Exempt towns (n = 19 172)^b^	Work requirements eliminated (n = 9626)	Work requirements remained (n = 12 146)	Exempt towns (n = 21 772)^b^
Age group,y						
25-29	2914 (34.3)	3779 (35.4)	6693 (34.9)	1611 (16.7)	1672 (13.8)	3283 (15.1)
30-39	2840 (33.5)	3642 (34.1)	6482 (33.8)	4296 (44.6)	5170 (42.6)	9466 (43.5)
40-49	2736 (32.2)	3261 (30.5)	5997 (31.3)	3719 (38.6)	5304 (43.7)	9023 (41.4)
Race and ethnicity^c^						
Hispanic	534 (6.3)	1424 (13.3)	1958 (10.2)	1129 (11.7)	3307 (27.2)	4436 (20.4)
Non-Hispanic Black	308 (3.6)	1052 (9.8)	1360 (7.1)	397 (4.1)	1237 (10.2)	1634 (7.5)
Non-Hispanic White	7458 (87.8)	7802 (73.0)	15 260 (79.6)	7671 (79.7)	6741 (55.5)	14 412 (66.2)
Other	190 (2.2)	404 (3.8)	594 (3.1)	429 (4.5)	861 (7.1)	1290 (5.9)
Sex						
Female	3312 (39.0)	4164 (39.0)	7476 (39.0)	7016 (72.9)	8944 (73.6)	15 960 (73.3)
Male	5178 (61.0)	6518 (61.0)	11 696 (61.0)	2610 (27.1)	3202 (26.4)	5812 (26.7)
Resident in rural town^d^	845 (10.0)	1587 (14.9)	2432 (12.7)	871 (9.0)	1539 (12.7)	2410 (11.1)
Enrolled in SNAP in before work requirements^e^	2848 (33.5)	3046 (28.5)	5894 (30.7)	4247 (44.1)	4671 (38.5)	8918 (41.0)
Unhoused	289 (3.4)	452 (4.2)	1049 (5.5)	14 (0.1)	31 (0.3)	79 (0.4)
≥1 Chronic condition	3462 (40.8)	3916 (36.7)	7545 (39.4)	4120 (42.8)	4855 (40.0)	9813 (45.1)

Abbreviation: SNAP, Supplemental Nutrition Assistance Program.

^a^ Postmatching sample of adult Medicaid beneficiaries aged 25 to 49 years who were eligible for SNAP. Only 33 targeted (<.0.1%) and 26 untargeted (<0.1%) beneficiaries in work requirement towns were dropped during matching. To improve the comparability of groups, individuals in exempt towns were matched with individuals in work requirement towns by age, sex, race and ethnicity, and residence in a rural town.

^b^ Due to matching 1:1 with replacement, there were 13 685 unique targeted and 16 015 unique untargeted beneficiaries in exempt towns.

^c^ Self-reported by enrollees from eligibility data. Other included Asian, Native American or Alaska Native, or Native Hawaiian or Other Pacific Islander.

^d^ Based on the rural definition from the Connecticut State Office of Rural Health, towns were encoded as rural if they had a population of 10 000 or less and a population density of 500 or fewer people per square mile.

^e^ The full 9-month period prior to SNAP benefits being put at risk. This includes July through December 2015, plus the 3-month time limit imposed by work requirements from January through March 2016.

## Results

The study included 81 888 enrollees as of March 2016, the month before SNAP work requirements put benefits at risk. Work requirements were associated with a mean reduction in enrollment of 5.4 (95% CI, 4.6-6.3) percentage points over the 9 months following implementation. Reversal of the policy was not associated with a change in enrollment (mean difference, 1.2 [95% CI, −0.1 to 2.6] percentage points) over the subsequent 9 months in towns where work requirements were reversed (Figure).

**Figure.**
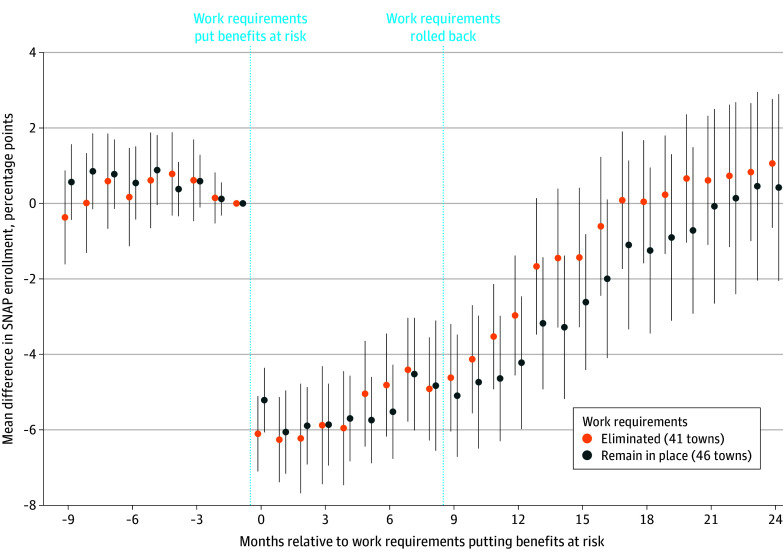
Triple-Difference Event Study of Supplemental Nutrition Assistance Program (SNAP) Enrollment Before and After the Work Requirements Policy Among Treated Able-Bodied Adults Without Dependents vs Untreated Parents and Caregivers in Affected vs Exempted Towns Time was measured relative to the month prior to work requirements putting SNAP benefits at risk in March 2016. SEs were clustered at the town level based on enrollee's town in that month. The policy was reversed starting in January of 2017. Error bars indicate 95% CIs.

## Discussion

While work requirements in SNAP were associated with immediate coverage losses, removing work requirements was not associated with a change in enrollment. The enrollment patterns were similar in towns with and without a policy reversal. One potential explanation is that households automatically lost coverage when work requirements were imposed via procedural terminations but needed to be aware of a policy that eliminated work requirements and required a manual process of reapplying.

A limitation is there was possibility of spillover effects of work requirements for individuals in exempt towns, but prior research^2^ suggests this is unlikely. Consistent with findings in Medicaid,^5^ we found that coverage losses from work requirements largely dissipated 9 months after reversal. Our results suggest this was not driven by the targeted group reenrolling but by secular declines in the nontargeted comparison cohorts. Our study highlights that work requirement implementation may have lasting effects that are difficult to reverse.

## Notes

Supplement 1. **eFigure 1.** Connecticut Towns by Work Requirement Status

Supplement 2.
Data Sharing Statement
