# Supplementary material for: SNAP Work Requirements Reversal and Program Enrollment
Source: JAMA Health Forum. 2025 May 30;6(5):e251587. doi: 10.1001/jamahealthforum.2025.1587 (PMC12125636; doi:10.1001/jamahealthforum.2025.1587)
Supplement: Supplement 1. — eFigure 1. Connecticut Towns by Work Requirement Status [file jamahealthforum-e251587-s001.pdf]

Supplemental Online Content

Factor H, Wallace J, Lavallee M, Lollo A, Ndumele CD. SNAP work requirements reversal and program enrollment. *JAMA Health Forum.* Published online May 30, 2025. doi:10.1001/jamahealthforum.2025.1587

eFigure 1. Connecticut Towns by Work Requirement Status

This supplemental material has been provided by the authors to give readers additional information about their work.

© 2025 Factor H et al. *JAMA Health Forum*.

eFigure 1. Connecticut Towns by Work Requirement Status

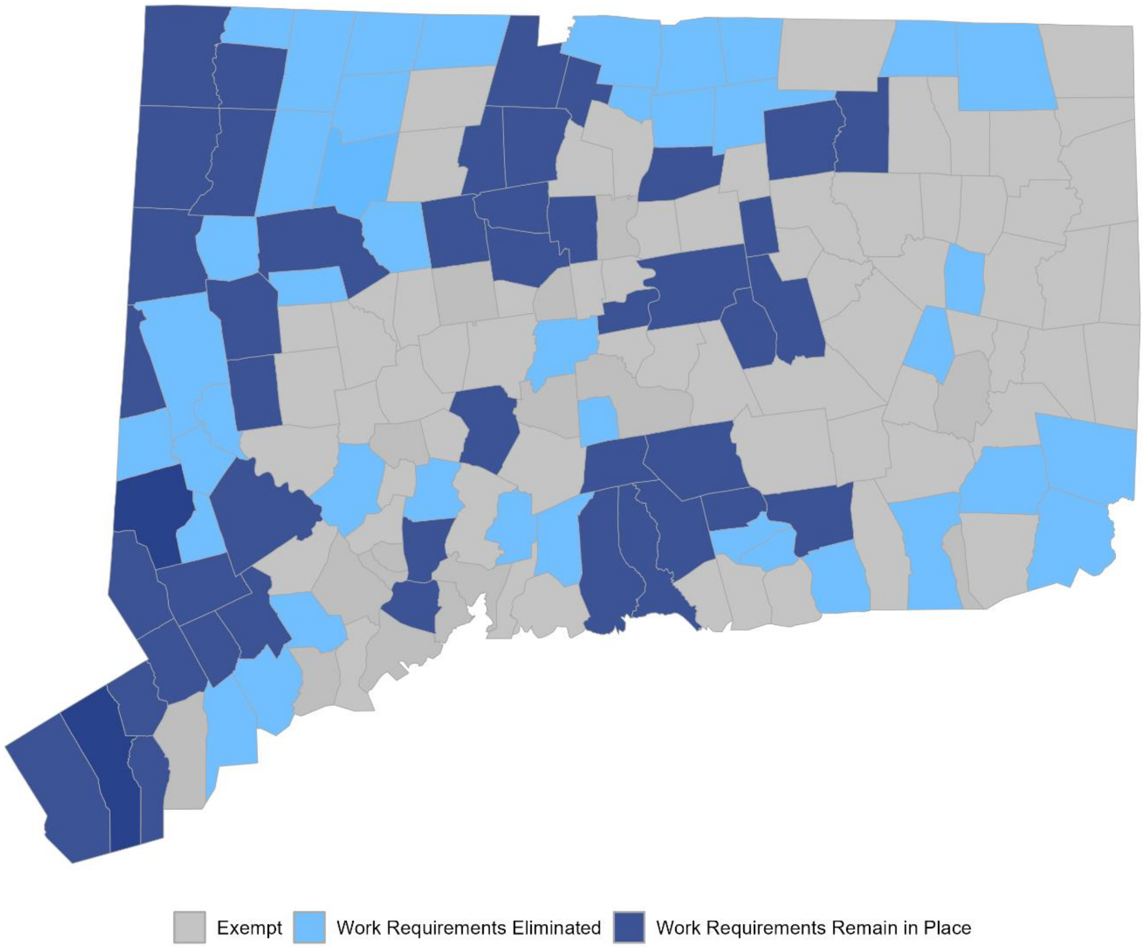

Notes: Figure 1 illustrates the 169 towns in Connecticut and their work requirement status throughout the study period. Towns shaded grey were exempt from work requirements for the entire period. Dark blue towns had work requirements enacted on January 1, 2016 which remained in place throughout the rest of the period. Light blue towns had work requirements enacted on January 1, 2016 which were subsequently eliminated on January 1, 2017

© 2025 Factor H et al. *JAMA Health Forum*.
