# Supplementary material for: SNAP Work Requirements Reversal and Program Enrollment
Source: JAMA Health Forum. 2025 May 30;6(5):e251587. doi: 10.1001/jamahealthforum.2025.1587 (PMC12125636; doi:10.1001/jamahealthforum.2025.1587)
Supplement: Supplement 2. — Data Sharing Statement [file jamahealthforum-e251587-s002.pdf]

Data Sharing Statement

Factor. SNAP Work Requirements Reversal and Program Enrollment. *JAMA Health Forum*. Published May 30, 2025. doi:10.1001/jamahealthforum.2025.1587

Data
Data available: No

Additional Information
Explanation for why data not available: Proprietary Administrative Enrollment and Claims Data. Samples of the software code used to produce the final results in our manuscript will be made available in a public repository at https://github.com/Yale-Medicaid.
